# Supplementary material for: Genomic and Temporal Analysis of Deletions Correlated to qRT-PCR Dropout in N Gene in Alpha, Delta and Omicron Variants
Source: Viruses. 2023 Jul 26;15(8):1630. doi: 10.3390/v15081630 (PMC10458892; doi:10.3390/v15081630)
Supplement: Supplementary file 1 [file viruses-15-01630-s001.zip › Table S1.docx]

TableS1: the presence of deletions was checked and confirmed by reporting the Deptht of Coverage (the exact number of reads mapping in a specific base position) and the Variant Calling (the percentage of reads carrying the mutation).

| **Sample number** | **DC*** | **VF**** | **Mutation** |
| --- | --- | --- | --- |
| 1 | 7374 | 99.3 | Pro207_Ala208del |
| 2 | 8999 | 99.4 | Pro207_Ala208del |
| 3 | 10035 | 99.9 | Pro207_Ala208del |
| 4 | 20016 | 99.5 | Pro207_Ala208del |
| 5 | 4253 | 99.6 | Pro207_Ala208del |
| 6 | 4843 | 99.8 | Pro207_Ala208del |
| 7 | 2506 | 99.8 | Pro207_Ala208del |
| 8 | 5801 | 99.7 | Pro207_Ala208del |
| 9 | 4720 | 99.8 | Pro207_Ala208del |
| 10 | 6935 | 99.8 | Pro207_Ala208del |
| 11 | 11198 | 100 | Arg203_Ser206del |
| 12 | 4578 | 99.7 | Pro207_Ala208del |
| 13 | 5351 | 99.9 | Pro207_Ala208del |
| 14 | 1993 | 99.4 | Pro207_Ala208del |
| 15 | 4119 | 99.9 | Pro207_Ala208del |
| 16 | 12644 | 99.9 | Ala208_Arg209del |
| 17 | 21423 | 100 | Ala208_Arg209del |
| 18 | 5923 | 99.9 | Pro207_Ala208del |
| 19 | 17321 | 99.9 | Pro207_Ala208del |
| 20 | 6784 | 99.9 | Pro207_Ala208del |
| 21 | 6933 | 100 | Pro207_Ala208del |
| 22 | 9595 | 99.9 | Pro207_Ala208del |
| 23 | 11752 | 100 | Pro207_Ala208del |
| 24 | 5024 | 99.7 | Pro207_Ala208del |
| 25 | 8191 | 99.9 | Pro207_Ala208del |
| 26 | 20505 | 99.9 | Ala208_Arg209del |
| 27 | 20805 | 100 | Pro207_Ala208del |
| 28 | 4584 | 99.9 | Ala208_Arg209del |
| 29 | 4735 | 82.7 | Ala208_Arg209del |
| 30 | 34144 | 99.8 | Ala208_Arg209del |
| 31 | 6970 | 99.6 | Ala208_Arg209del |
| 32 | 5071 | 99.9 | Gly214_Gly215del |
| 33 | 14343 | 99.9 | Gly214_Gly215del |
| 34 | 12351 | 100 | Gly214_Gly215del |
| 35 | 15677 | 99.9 | Gly214_Gly215del |
| 36 | 34168 | 99.9 | Gly214_Gly215del |
| 37 | 18008 | 99.9 | Gly214_Gly215del |
| 38 | 9058 | 99.9 | Gly214_Gly215del |
| 39 | 18604 | 99.6 | Gly214_Gly215del |
| 40 | 7415 | 99.9 | Gly214_Gly215del |
| 41 | 3909 | 98.3 | Gly214_Gly215del |
| 42 | 10075 | 98.3 | Gly204_Arg209del |
|  | 15600 | 99.9 | Glu31_Ser33del |
| 43 | 8919 | 99.9 | Glu31_Ser33del |
|  | 5270 | 99.5 | Ala208_Arg209del |
| 44 | 12517 | 99.7 | Glu31_Ser33del |
|  | 8465 | 99.7 | Ala208_Arg209del |
| 45 | 3783 | 100 | Glu31_Ser33del |
|  | 2662 | 99.9 | Arg209_Gly212del |
| 46 | 5764 | 100 | Arg203_Ala208del |
|  | 5995 | 100 | Glu31_Ser33del |
| 47 | 8758 | 99.7 | Arg209_Met210del |
|  | 24788 | 99.8 | Glu31_Ser33del |
| 48 | 35250 | 100 | Gly214_Gly215del |
|  | 38026 | 99.9 | Glu31_Ser33del |
|  |  |  |  |

* DC: Depth of Coverage.

** VF: Variant Fraction.
