# Supplementary figures and images for: Genomic and Temporal Analysis of Deletions Correlated to qRT-PCR Dropout in N Gene in Alpha, Delta and Omicron Variants
Source: Viruses. 2023 Jul 26;15(8):1630. doi: 10.3390/v15081630 (PMC10458892; doi:10.3390/v15081630)

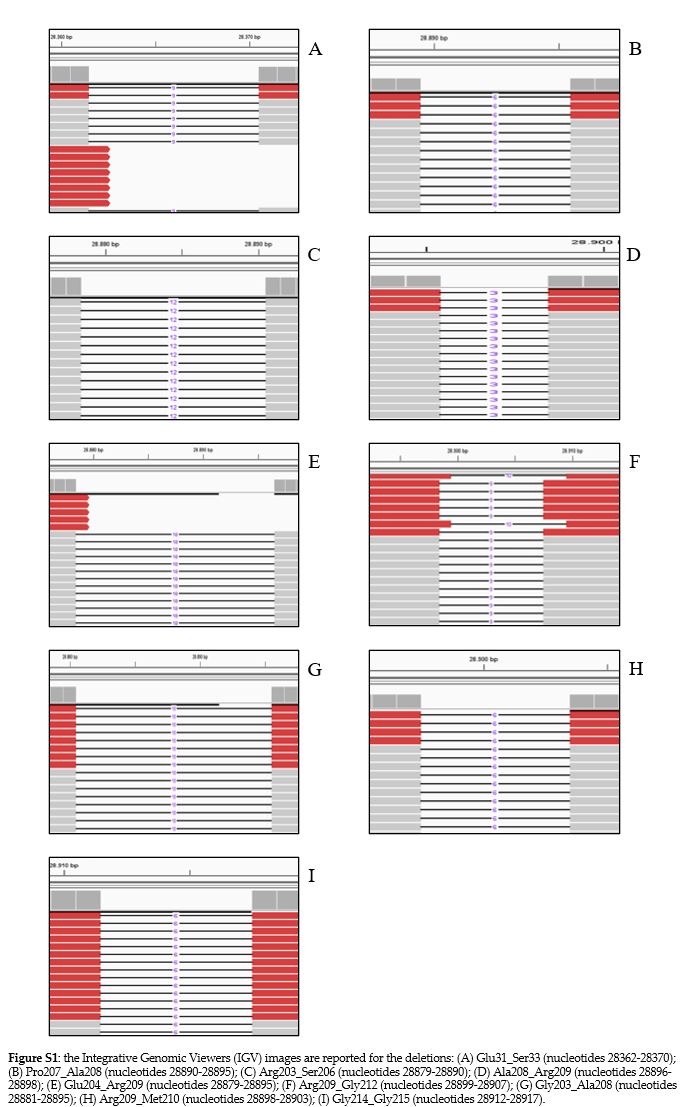

Supplement: Supplementary file 1 [file viruses-15-01630-s001.zip › Figure S1_new.JPG]

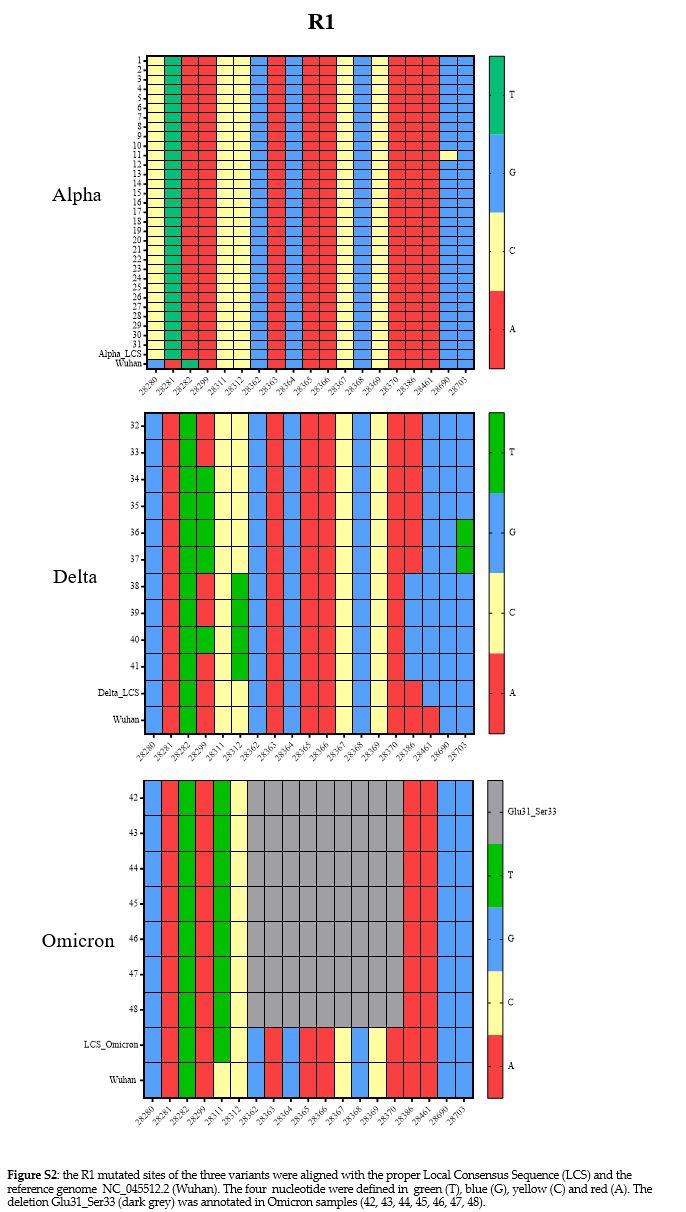

Supplement: Supplementary file 1 [file viruses-15-01630-s001.zip › Figure S2_new.JPG]

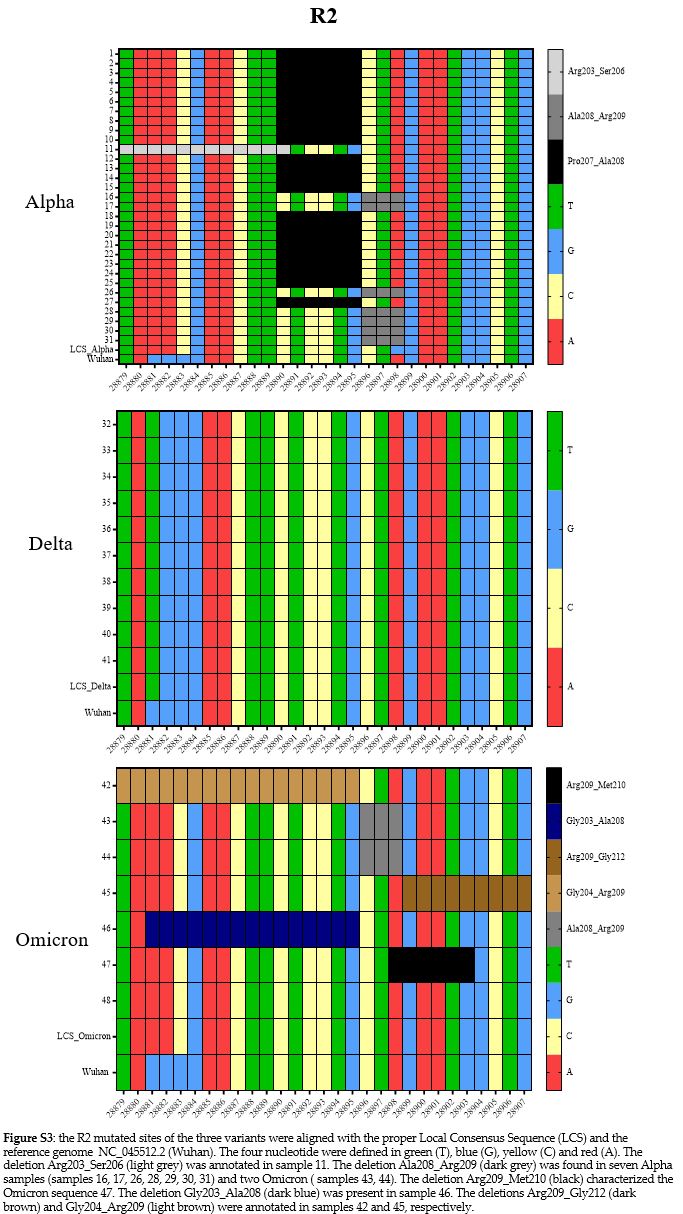

Supplement: Supplementary file 1 [file viruses-15-01630-s001.zip › Figure S3_new.JPG]

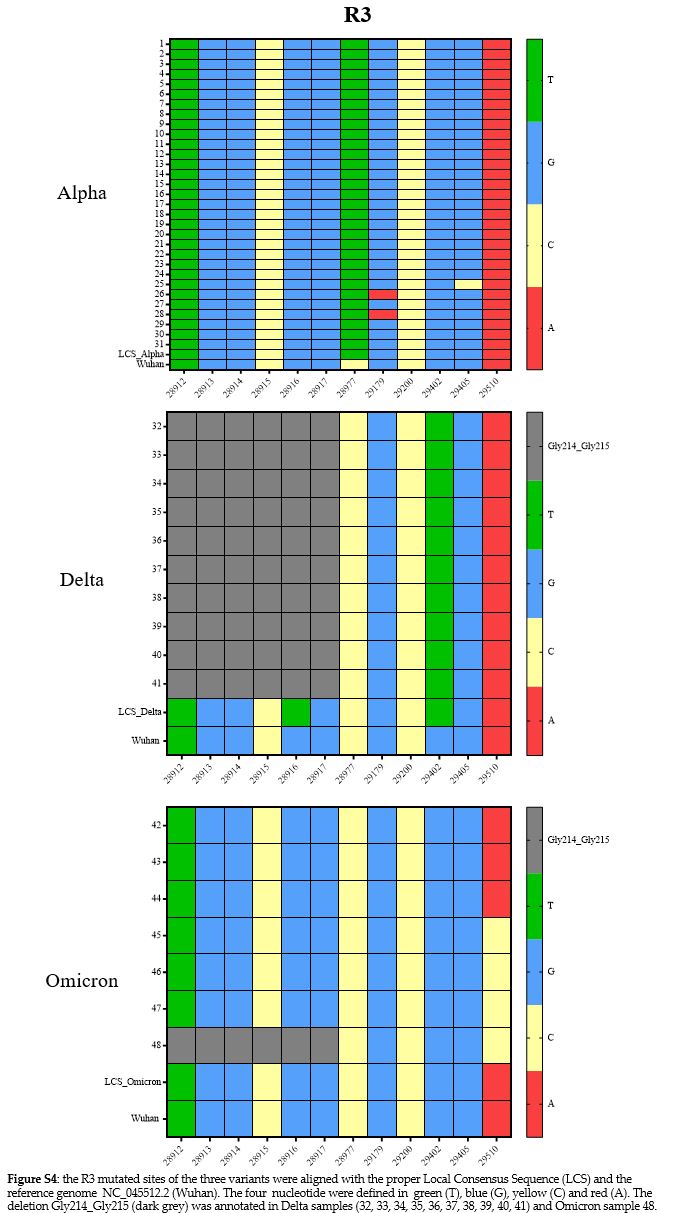

Supplement: Supplementary file 1 [file viruses-15-01630-s001.zip › Figure S4_new.JPG]
